# Electrophysiological evaluation of Wolff-Parkinson-White Syndrome

**Published:** 2002-10-01

**Authors:** Beatrice Brembilla-Perrot

**Affiliations:** Cardiology, CHU of Brabois, 54500 Vandoeuvre, France

**Keywords:** Wolff-Parkinson-White syndrome, sudden death, Electrophysiologic study

## Abstract

Sudden death might complicate the follow-up of symptomatic patients with the Wolff-Parkinson-White syndrome (WPW) and might be the first event in patients with asymptomatic WPW. The risk of sudden death is increased in some clinical situations. Generally, the noninvasive studies are unable to predict the risk of sudden death correctly . The electrophysiological study is the best means to detect the risk of sudden death and to evaluate the nature of  symptoms. Methods used to define the prognosis of WPW are well-defined. At first the maximal rate of conduction through the accessory pathway  is evaluated; programmed atrial stimulation using 1 and 2 extrastimuli delivered at different cycle lengths is then used to determine the accessory pathway refractory period and to induce a supraventricular tachycardia. These methods should be performed in the control state and repeated in adrenergic situations either during exercise test or more simply during a perfusion of small doses of isoproterenol. The induction of an atrial fibrillation with rapid conduction through the accessory pathway (> 240/min in control state, > 300/min after isoproterenol) is the sign of a form of WPW at risk of sudden death.

## Introduction

At the time of the curative treatment of Wolff-Parkinson-White syndrome (WPW) by radiofrequency ablation [1,2], it is particularly important to detect the forms at risk of sudden death. Sudden death in WPW syndrome is related to the degeneration of atrial fibrillation with high ventricular rates into ventricular fibrillation.

## Natural History Of Wolff-Parkinson-White Syndrome

The exact risk of patients with the WPW syndrome to develop this complication is not known. It was relatively high in old studies in symptomatic patients [3,4] (1.5%), but was low in asymptomatic patients [5-7]. The most recent study [8] reports a sudden cardiac death risk of 0.02%/patient/year. Previous studies [9,10] have shown that ventricular fibrillation can be the first event of the Wolff-Parkinson-White syndrome. In the studies of Turner Montoya [9] and Timmermans [10], ventricular fibrillation was the first manifestation of the WPW syndrome in  26% and 53% of their series respectively. The risk is low, but these sudden deaths are very regrettable; concerning patients with normal hearts except for the WPW, which is easy to cure. Therefore, the detection of patients with WPW at risk of sudden death is fundamental. The studies in patients with aborted sudden death indicated that in most of these patients an atrial fibrillation with rapid conduction through the accessory pathway is the main finding. How to detect these patients?

## Methods of Evaluation

The pattern of WPW syndrome on 12 lead surface ECG, the permanent or intermittent feature are not specific, although posteroseptal location and permanent forms are more frequently noted in patients at risk of rapid arrhythmias [9,10].

 Several non invasive studies were proposed but their diagnostic value is low :

- The abrupt disappearance of WPW syndrome during an exercise stress testing was proposed as a sign of accessory pathway with long refractory period [11-13]; however the abrupt disappearance syndrome  is observed rarely, even in patients without risk of severe arrhythmias ; moreover, it can be noted in patients with a short accessory period refractory period [14] as well.

- Pharmacological tests  were also proposed : the disappearance of the pattern of WPW syndrome was reported as a sign of accessory pathway with long refractory period [15]; however, many false positive tests were reported [16].

Electrophysiologic study appears to be the most reliable method to establish the prognosis of WPW syndrome [17]

## Methods of Electrophysiologic Study in WPW

Patients might be studied by transesophageal route [18,19] or intracardiac route. The first route is indicated in asymptomatic patients and the second in symptomatic patients to perform the catheter ablation of the accessory pathway in a second time ; the advantages of esophageal route is that the patient is not hospitalized and is leaving hospital after electrophysiologic study. Those with induced atrial arrhythmias are monitored until sinus rhythm is restored. It is generally not necessary to hospitalize these patients.

**Figure 1:**
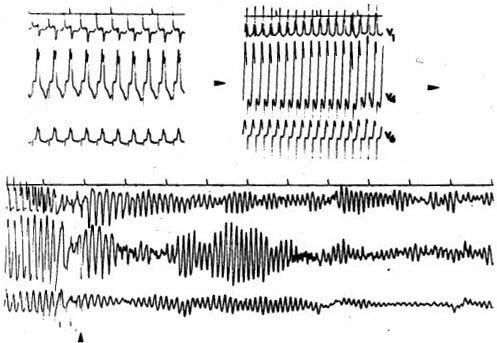
Direct induction of a ventricular fibrillation by esophageal atrial pacing at progressively increasing  rates in an asymptomatic young man

It should be noted that all electrophysiologic studies in WPW syndrome might be dangerous and should be performed with an external defibrillator ready to be used, because a ventricular fibrillation can be induced in asymptomatic or symptomatic patients [20] (Figure 1).

Surface electrocardiograms and esophageal electrogram are simultaneously recorded on paper at speeds of 25 or 100 mm/sec. Cardiac stimulation is performed with a programmable stimulator which is connected to a pulse amplifier that can deliver pulses at width of 16 ms with a 29 mA output in the case of esophageal stimulation. For a simple electrophysiological study, only one catheter is needed : a bipolar silicone esophageal lead or one bipolar intracardiac catheter. A multipolar catheter electrode is  used only for the mapping of left atrium in patients who need a catheter ablation of the accessory pathway.

**Figure 2:**
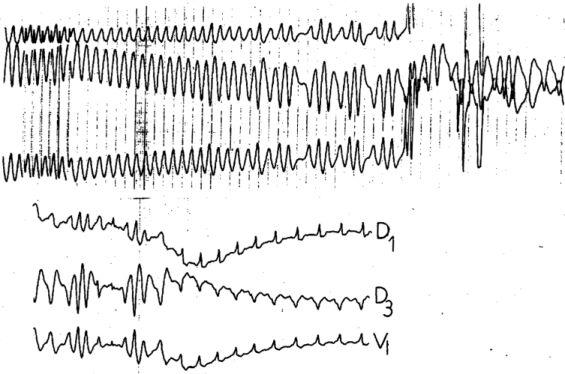
Rapid conduction through the accessory pathway during atrial pacing (> 300b/min) and induction of an atrial fibrillation with very rapid conduction through the accessory pathway (300 b/min or cycle length 200 ms) = electrophysiological signs of a form at risk of sudden death

The classical protocol is as follows:

- Incremental atrial pacing is performed until second degree atrioventricular block occurs. The maximal rate of 1/1 conduction trough the accessory should be noted.

- Programmed atrial stimulation at  basic cycle lengths of 600 ms and 400 ms with the introduction of one and two extrastimuli is performed : the disappearance of WPW syndrome indicates the accessory pathway refractory period. The method  is also used to induce a supraventricular tachycardia, generally an orthodromic tachycardia, rarely an antidromic tachycardia or an atrial tachycardia or fibrillation.

- These data should be studied under adrenergic situations, except in patients who have a risk of sudden death in control state. Two methods are used : atrial pacing might be repeated during an exercise testing  [21,22] which is the most physiological situation, but difficult to perform. Many authors prefer the infusion of isoproterenol, a beta-adrenergic drug and which is more simple to use during an electrophysiologic study. Isoproterenol (0.02 to 1 μg.min-1) is infused to increase the sinus rate to at least 130 beats. min-1 and the pacing protocol is repeated [21-23]. At the end of the protocol, using the following definitions, the prognosis of WPW syndrome can be established. These are arrived at using   the electrophysiological characteristics of patients with WPW and aborted sudden death.

- Sustained atrial fibrillation or reciprocating tachycardia is defined as a tachycardia that is longer than 1 minute. The exact duration of induced tachycardia to be considered as pathological is still controversed and varies from 30 sec up to 5 minutes [24].

- Conduction over the accessory atrioventricular connection is evaluated by the measurement of the shortest atrial cycle length at which there is 1 to 1 conduction over the accessory connection and the shortest atrial tachycardia cycle length at which there is 1 to 1 conduction over the accessory connection.

- The Wolff-Parkinson-White syndrome is considered as representing a risk of sudden death when the following association is observed : sustained atrial fibrillation is induced and the shortest RR interval between preexcited beats is < 250 ms in the control state in adults, < 220 ms in children [25] or < 200 ms during isoproterenol infusion  [26] (Figure 2).

## General Results of Electrophysiological Studies in WPW Syndrome

**Figure 3:**
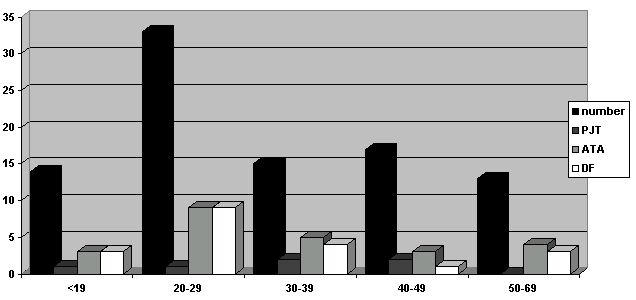
representation of the data of entire population according to the age : - in black : total number of patients - in dark grey : number of induced reciprocating tachycardia - in light grey : number of induced atrial tachyarrhythmia - in white : number of dangerous form of WPW syndrome

- The exact nature of the prexcitation syndrome is assessed. Most of the WPW syndrome are related to a atrioventricular accessory connection or Kent bundle : the degree of prexcitation increases during premature atrial stimulation until the refractory period of accessory pathway is reached, because the conduction time does not change in accessory pathway with the shortening of atrial cycle length while it increases in the AV node. Rarely the WPW syndrome is related to a nodoventricular accessory pathway or Mahaim bundle and the degree of preexcitation remains unchanged during premature atrial stimulation.

- The accessory pathway refractory period depends on the driven cycle length. Refractory period of the accessory pathway decreases as the driven cycle length shortens.

- Beta adrenergic stimulation results in shortening of the anterograde refractory period of the accessory pathway  and an increase in ventricular rates during atrial pacing and atrial fibrillation [27]. Isoproterenol test was also previously used to verify the efficacy of antiarrhythmic drug before the era of catheter ablation of accessory pathway. The loss of efficacy of some antiarrhythmic drugs was demonstrated after isoproterenol administration  [28].

- Atrial fibrillation is easily induced during intracardiac studies by salvos of rapid atrial stimulation and is not specific [29]. The induction of an atrial fibrillation by intracardiac programmed stimulation is obtained in 27 % [21,30], 41 %   [24] or 56 %  [28], according to various studies in asymptomatic patients and in 75 % of patients with only documented reentrant tachycardia [24]; atrial fibrillation is induced in 95 % [29] of those with documented atrial fibrillation [24]. The important variations of the incidence of induced atrial fibrillation depends on the technique of programmed stimulation, on the interpretation of the duration of induced arrhythmia and on the use of isoproterenol infusion or other means to reproduce the effects of adrenergic stimulation. In our experience, the induction of an atrial fibrillation during transesophageal pacing has a best clinical significance  [29] : the induction is rarer, from 10 to 30 % according to the age in patients without documented atrial fibrillation and remained sensitive to induce atrial fibrillation in those with documented atrial fibrillation (95 %). The incidence of induction of atrial fibrillation also depends on the presence of an associated heart disease and the age of the patient : the induction of atrial fibrillation is rarely noted in children younger than 10 years, is induced in 20 % of teenagers and adults without heart disease and becomes relatively frequent in elderly (31%)  [31].

- Ventricular tachyarrhythmias also are easily induced in asymptomatic or symptomatic patients by programmed ventricular stimulation and are not specific in patients with WPW syndrome : the induction of a ventricular fibrillation is noted in 4 % of WPW syndrome and the induction of nonsustained multiform ventricular tachycardia in 37 % of them [32].

- Antidromic tachycardia which is a reciprocating tachycardia using the accessory pathway for the anterograde conduction and the normal AV conduction system for retrograde conduction, is a rare finding (5%), more frequently noted in young patients with a good retrograde normal VA conduction or in patients with several accessory pathways and seems more frequent in patients at risk of rapid arrhythmias.

- Orthodromic tachycardia which is a reciprocating tachycardia using the normal AV conduction system for the anterograde conduction and the accessory pathway for the retrograde conduction, is rarely induced in asymptomatic patients (< 10%) [33,34], but represents the most frequent tachycardia of symptomatic patients complaining tachycardia and palpitations (90 %) [24].

- The incidence of forms considered at risk of rapid arrhythmias is similar in patients with symptomatic and asymptomatic patients and concerns 10 % of the total population with WPW syndrome  [33,34], independent of the age of the patient [31] (Figure 3). However the exact clinical significance of the electrophysiological form at risk of sudden death in asymptomatic patients remains controversial. Previous studies using the intracardiac evaluation of WPW syndrome noted a low incidence of adverse events during a mean follow-up of 4 years [35,36]. Actually, it is no longer possible to evaluate the exact clinical significance of these forms considered at risk of sudden death, because of the important development of the techniques of catheter ablation of accessory pathway.

Moreover, the presence of syncope does not increase the chance to find a potentially dangerous form in adults [37]; in young patients (< 25 years) the significance of syncope seems different and associated with occurrence of atrial fibrillation with a rapid ventricular response (sensitivity 64 %, specificity 100 %)  [38].

## Indications of Electrophysiological Studies in WPW

**Figure 4:**
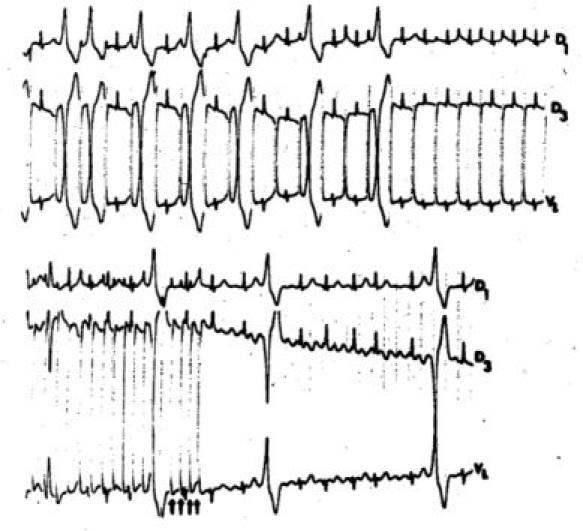
Slow conduction through an accessory pathway and induction by very rapid atrial pacing of an atrial fibrillation conducted through the normal AV conduction system = electrophysiological signs of a benign WPW syndrome without risk of sudden death

- The indications of electrophysiological study are now large in symptomatic patients to perform in a second time the catheter ablation of patients complaining frequent sustained tachycardias. The study should be performed by catheterisation.

- In patients with syncope, but no documented tachycardia, electrophysiological study is necessary and might be performed by transesophageal route because the role of the accessory pathway in the occurrence of syncope remains rare in adults [37].

- In patients who have a documented rapid or syncopal atrial fibrillation, electrophysiological study is not indicated, because direct catheter ablation of the accessory pathway is recommended. The location of the Kent bundle is easier in sinus rhythm and the induction of an atrial fibrillation should be avoided.

- In asymptomatic patients, the indications of electrophysiolgical study are more debatable [39]. At first if the study is indicated, esophageal route should be preferred, because the probability to find a form at potential risk of sudden death remains rare (10 %). The main interest is to allow the patients in 90 % of cases to continue their activities in presence of an electrophysiological form without signs of risk of rapid atrial arrhythmias (Figure 4).

Some indications of a systematic electrophysiological are actually recommended :

1) most of sudden deaths have the peculiarity to occur during exercise [40]. Because of the important development of sports from the infancy to the elderly, it is important to detect those patients with WPW at risk of sudden death who practice a sportive activity; the indication generally, begins after 10 years, because the risk of induction of a rapid atrial fibrillation is very small and the level of sport still low. In adults only those who practice a sport at a high level (for example bicycle) are studied. The competitive athlete should be studied in all ranges of age. The indications are also recommended in professions with a high level of sportive activity (policemen, soldier, fireman…)

2) the second indication is the detection of a WPW syndrome in a patient with high responsibility profession such as professional pilot (plane, truck, bus, train)

While these indications are largely in teenagers and adults less than 40 years of age, the indications in children or elderly are more controversial :

- in children, the conduction in accessory pathway and normal AV conduction system are more rapid, probably without  a clinical significance : in the study of Bromberg  [25] a cycle length < 220 ms in basal state is considered at risk of severe arrhythmias in children < 18 years. In adults, the value of <250 ms is taken as a sign of a dangerous form. Moreover, the increase in conduction velocity in accessory pathways was reported in children and the disappearance of the Wolff-Parkinson-White syndrome can be expected, but this is inconstant and not predictible [41]. Therefore, because some sudden deaths as the first event were reported in children [25,33], the indications should be liberal in children who are competitive athletes and in all children above the age of 10 years.

- in elderly, the shortest atrial pacing cycle length with 1:1 anterograde conduction via the bypass tract increased progressively with age [42-44]. However, the propensity for atrial fibrillation was shown to be higher in older patients compared to younger patients [45]. While the exact mechanism is uncertain, degenerative changes associated is the most commonly proposed mechanism and the dispersion of atrial refractoriness increases progressively with age [45].The risk to have a severe arrhythmia as the first manifestation of WPW syndrome in an old patient was previously reported [46]. High level sportive activity is rare in elderly, but other causes for adrenergic tone increase might be encountered : for example, an important surgery was the cause of the development of a ventricular fibrillation in a 72 year old asymptomatic patient in our experience.

Therefore, because of the increase of the sport in all ranges of age and particularly in young children or after 60 years, the risk of occurrence of a potentially severe arrhythmia in an asymptomatic WPW patient should be not underestimated. The reliability and the simplicity of transesophageal study in WPW permits easy detection of forms at risk of severe arrhythmia.

In conclusion, electrophysiological study is the best means to define the prognosis of a patient with the WPW syndrome. The study is easily performed by the transesophageal route. The indications should be large to avoid the misdiagnosis of a form at risk of rapid arrhythmias. This dangerous form is relatively rare in  asymptomatic patients or symptomatic patients with unexplained syncope. Most of these patients (>85%) would be allowed to continue their activities, without specific treatment, because they have a benign form of Wolff-Parkinson-White syndrome. In remaining patients, the development of the curative treatment of this disease by radiofrequency application on the accessory pathway [46] permits to offer the possibility to this patient to continue the sport or some professions with stress or exercise. However, if the data of electrophysiological study are clear and admitted in all studies, their consequences are still debatable : radiofrequency current ablation of asymptomatic patients with the Wolff-Parkinson-White syndrome is controversial and requires other studies with randomized series comparing untreated patients and patients treated by radiofrequency ablation of the accessory pathway.
